# La prise en charge de la douleur chez l'enfant cancéreux

**DOI:** 10.11604/pamj.2015.21.319.6690

**Published:** 2015-08-28

**Authors:** Mohamed Moukhlissi, Malika Aitidir, Imane Bouamama, Khadija Maani, Jamila Hachim

**Affiliations:** 1 Département de Pédiatrie, Unité d'Hémato-Oncologie, CHU Casablanca, Maroc

**Keywords:** Douleur, Enfant, cancer, traitement de la douleur, pain, child, cancer, pain management

## Abstract

C'est une étude prospective étalée sur une année (Juin 2007 à Mai 2008) portant sur une série de 140 malades (84 garçons et 56 filles), hospitalisés à l'unité d'hémato-oncologie du service de pédiatrie III à l'hôpital d'enfant de Casablanca. L'objectif de l’étude est de reconnaître la douleur chez l'enfant, l’évaluer en fonction des échelles internationales, et d’ assurer sa prise en charge globale en rapportant l'expérience du service. L’évaluation de la douleur a concerné différentes pathologies cancéreuses: quatre vingt enfants avaient des leucémies aigues lymphoblastiques (57%), 24 enfants présentaient des lymphomes (17%), 10 enfants avaient des neuroblastomes (7%) et 10 autres des néphroblastomes (7%). Parmi les 140 malades évalués, 100 présentaient des douleurs et qui ont fait l'objet de notre étude, donc on peut estimer la prévalence de la douleur ressentie au cours de l'hospitalisation à 71,4%. Plusieurs types d’échelles en fonction de l’âge de l'enfant, et de sa capacité à exprimer par lui-même, ou non, sa douleur, ont été mises à la disposition pour une mesure chiffrée et reproductible mais aussi une base à l'adaptation du traitement antalgique et pour assurer un suivi évolutif sous traitement. Le Traitement anticancéreux reçu par les malades à but curatif: dix malades soit 7,2% ont bénéficié d'une chirurgie curative et une chimiothérapie, cent vingt cinq enfants (89,2%) ont reçu une chimiothérapie seule et cinq malades (3,6%) ont bénéficié d'une association chirurgie, chimiothérapie et radiothérapie. La douleur ressentie avait en lien direct avec la maladie cancéreuse chez 30% des cas (30 enfants), secondaire au traitement anticancéreux chez 30% (30 enfants), et elle était surtout induite par des actes invasifs à visée diagnostique ou thérapeutique chez 40% des cas (40 enfants). Le traitement antalgique a été instauré en fonction de l’évaluation clinique: 67% des patients ont nécessité des antalgiques niveaux I ou II, l'usage des antalgiques niveaux III a eu lieu dans 20% des cas alors que dans 13% des cas le seuil de la douleur n'exigeaient aucun traitement. La prise en charge de la douleur est une étape fondamentale dans le traitement du cancer de l'enfant, elle doit être évaluée dès l’étape diagnostique et surveillée tout au long du traitement.

## Introduction

La douleur chez l'enfant est un motif très fréquent de consultation en cancérologie, notion tellement subjective, et difficile à communiquer, a été longtemps négligée et sous traitée, ou du moins considérée comme inévitable, une réalité que les médecins ont longtemps niée du fait de l'immaturité neurologique et psychique du nourrisson et du petit enfant. Cependant l'avancée des connaissances neurophysiologiques acquises ces quinze dernières années a complètement invalidé ces dogmes médicaux qui ont permis pendant très longtemps de ne pas traiter la douleur chez l'enfant. Des études récentes ont permis de développer une véritable sémiologie de la douleur et d’établir des méthodes d’évaluation consensuelles. La reconnaissance de la douleur ainsi que sa prise en charge doit impliquer l'ensemble des acteurs des soins; elle nécessite initialement et obligatoirement d'admettre sa réalité chez l'enfant, elle ne doit plus reposer sur une appréciation intuitive des soignants. L'enfant cancéreux présentera un jour une expérience douloureuse, à cause de l'histoire naturelle de sa maladie, à cause des traitements, ou à cause des examens que nous devons lui faire subir pour s'assurer de sa guérison ou de l'efficacité des traitements. La réalité de la douleur doit être présente à l'esprit de toute l’équipe soignante et à tout moment, et doit être considérée comme une constante à rechercher, évaluer, prévenir et traiter au même titre que toute autre manifestation [1–3].

## Méthodes

Notre étude a été effectuée entre Juin 2007 et Mai 2008 à l'unité d'hémato-oncologie du service de pédiatrie III à l'hôpital d'enfant du centre hospitalier universitaire IBN ROCHD de Casablanca. L’étude a concerné tous les enfants présentant un cancer quels que soient le type histologique et le stade évolutif. Enfants hospitalisés au service afin d'assurer le suivi n'ayant pas eu de traitement antalgique avant l'hospitalisation. L’évaluation de la douleur est faite- en plus de l'interrogatoire, l'observation et l'examen clinique-par des échelles validées: Echelle Visuelle Analogique, Echelle Numérique, OPS (Objective Pain Scale)… Pour les enfants moins de six ans, on a utilisé des échelles d'hétéro évaluation, basée essentiellement sur l'observation, l'examen clinique et parfois l'interrogatoire avec les parents. Au-delà de 6 ans, la méthode utilisée était l'auto-évaluation. Trois questionnaires ont été élaborés de manière à évaluer au mieux la prise en charge de la douleur. Le premier questionnaire était destiné au patient permettant de recueillir les données suivantes: les variables épidémiologiques, les caractéristiques de la maladie cancéreuse, le traitement curatif reçu, l'existence ou non de la douleur, les différentes étiologies de la douleur, les localisations des douleurs, l'Intensité de la douleur, le retentissement de la douleur sur la qualité de vie, la prise en charge des douleurs et le suivi de l’évolution durant l'hospitalisation. Le second questionnaire a concerné le personnel soignant (médecins et infirmiers) prenant en charge les patients lors de leur hospitalisation, il a permis de recueillir des informations sur la méthode de la prise en charge de la douleur. Enfin le troisième questionnaire était un questionnaire évaluant le vécu du phénomène douloureux de l'enfant par les parents, et le degré de satisfaction concernant la qualité de la prise en charge.

## Résultats

### Données épidémiologiques

Cent quarante enfants ont été évalués entre Juin 2007 et Mai 2008 à l'unité d'hématologie et oncologie du service de pédiatrie III à l'hôpital d'enfants, au centre hospitalier universitaire IBN ROCHD de Casablanca. L’âge des enfants hospitalisés variait entre 2 ans et 15 ans. Plus de la moitié des malades étaient âgés de plus de 5 ans. Quatre vingt quatre enfants étaient de sexe masculin (60%), 56 de sexe féminin (40%) avec un sex-ratio garçon/ fille de 1.5.

### Caractéristiques de la maladie cancéreuse

**Catégories des pathologies rencontrées (Tableau 1):** quatre vingt enfants avaient une leucémie aigue lymphoblastique (57%), 24 enfants présentaient des lymphomes (17,2%), 10 enfants avaient des neuroblastomes (7,2%) et 10 autres des néphroblastomes (7,2%).

**Tableau 1:** Répartition des patients par pathologie

Maladie cancéreuse	Nombre de patients	Pourcentage (%)
Leucémie aigue lymphoblastique	80	57%
Leucémie aigue myéloblastique	5	3,6%
Lymphome malin	14	10%
Maladie d'Hodgkin	10	7,2%
Neuroblastome	10	7,2%
Néphroblastome	10	7,2%
Rhabdomyosarcome	5	3,6%
Sarcome d'Ewing	4	2,8%
Histiocytose	2	1,4%

**Localisations secondaires:** quatorze malades (10%) avaient des localisations secondaires. Cent vingt six malades (90%) n'avaient pas de métastases au moment de l’évaluation.

### Traitement anticancéreux reçu par les malades à but curatif

Dix malades soit 7,2% ont bénéficié d'une chirurgie curative associée à une chimiothérapie adjuvante, cent vingt cinq enfants (89,2%) ont reçu une chimiothérapie seule et cinq malades (3,6%) ont bénéficié d'une chirurgie, chimiothérapie et radiothérapie.

### La douleur à l'hôpital

**Figure 1:**
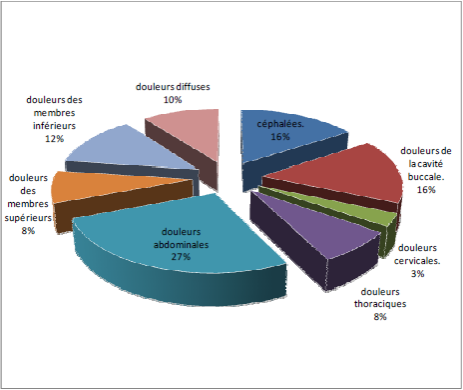
Les différentes localisations de la douleur

**Figure 2:**
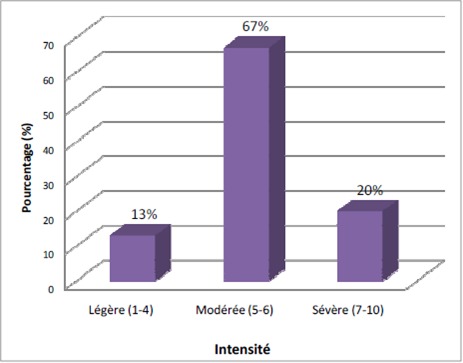
Pourcentage des patients en fonction de l'intensité de la douleur

Parmi les 140 malades évalués, 100 enfants présentaient des douleurs et qui ont fait l'objet de notre étude. L'incidence de la douleur au cours de l'hospitalisation est ainsi estimée à 71.4%. La durée moyenne de l'hospitalisation était de 5 jours avec des extrêmes allant de 2 à 22 jours. La douleur ressentie est en relation directe avec la maladie cancéreuse chez 30 enfants (30%), secondaire au traitement anticancéreux chez 30 enfants (30%) et induite par des actes invasifs à visée diagnostique ou thérapeutique chez 40 enfants (40%). La localisation de la douleur consiste à la détermination des zones douloureuses en utilisant le dessin du bonhomme de face et de dos (Figure 1). L’évaluation de l'intensité de la douleur chez 100 enfants a permis de conclure à une douleur légère dans 13% des cas, modérée dans 67%, et sévère dans 20% des cas Figure 2. La douleur a altèré significativement le sommeil chez 50% des enfants, elle avait un impact négatif sur l'humeur chez 66% et sur la capacité à jouer chez 74% des enfants.

### Prise en charge de la douleur

**Douleur liée a la maladie:** lTrente malades ont été pris en charge pour des douleurs liées à la maladie.

**Traitement antalgique:** vingt et un malades ont reçu des opioïdes faibles, 4 ont bénéficié des opioïdes forts, 3 malades n'ont reçu aucun traitement et 2 enfants ont été traités par des antalgiques non opioïdiques. Quatorze enfants ont reçu en plus du traitement antalgique des coanalgésiques.

**Evolution et efficacité:** l’évolution a été marquée par une très bonne amélioration chez 25 malades (83.3%) alors qu'on avait recours au changement du palier chez 5 malades (16.7%) dont l’évolution a été jugée favorable par la suite.

### Douleur liée aux actes iatrogènes

**Figure 3:**
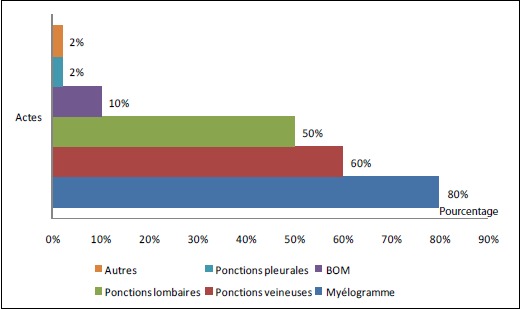
Répartition des actes douloureux

**Les actes aigus invasifs (Figure 3):** dans notre série la prévalence de la douleur liée aux actes invasifs est de 40%. Trente malades (30%) ont eu des ponctions veineuses un jour sur deux durant leur séjour à l'hôpital, 50 enfants (50%) ont eu des ponctions lombaires. Des myélogrammes ont été réalisés chez 80 malades (80%), des biopsies ostéo-médullaires (B.O.M) chez 10 enfants (10%). Deux enfants (2%) ont eu des ponctions pleurales et deux autres des ponctions d'ascite, un changement de pansement ou une biopsie ganglionnaire.

### La prise en charge de la douleur liée aux actes

**Le traitement antalgique:** Parmi les enfants qui ont souffert des douleurs liées aux soins, uniquement le tiers ont bénéficié de prévention par la crème EMLA et 10% par des antalgiques de palier I. Neuf enfants (9%) ont bénéficié d'une anesthésie générale pour la réalisation de la biopsie ostéo-médullaire (B.O.M). Trente quatre malades ont reçu des opioïdes faibles, 2 ont bénéficié des opioïdes forts, 4 malades n'ont reçu aucun traitement et aucun enfant n'a reçu des antalgiques non opioïdiques. vingt huit enfants ont reçu en plus du traitement antalgique des coanalgésiques.

**Evolution et efficacité:** l’évolution a été marquée par une très bonne amélioration chez 38 malades (95%), alors qu'on avait recours au changement du palier chez 2 malades (5%), dont l’évolution a été jugée favorable après 24 heures.

**Présence des parents lors des soins jugés générateurs des douleurs:** la majorité des enfants ayant présentés des douleurs liées aux actes aigus iatrogènes, étaient accompagnés de leurs parents lors du geste douloureux (64%).

**Information sur la nature du geste:** une enquête faite auprès du “TRIO” parent-enfant-équipe de soins, 40% des enfants ont été informé sur la potentialité de la douleur du geste, dans 30% des cas l'information a été communiquée directement aux parents, alors que chez 30% des malades aucune information concernant l'acte n'a été communiquée.

### Douleur liée au traitement anti-cancéreux

Trente malades ont été pris en charge pour des douleurs liées au traitement rapportées essentiellement à des mucites buccales dans 80% des cas.

**Le traitement antalgique:** dix sept malades ont reçu des opioïdes faibles, 7 ont bénéficié des opioïdes forts, 4 malades n'ont reçu aucun traitement et deux enfants ont été traités par des antalgiques non opioïdiques. Dix huit enfants ont reçu en plus du traitement antalgique, des co-analgésiques.

**Evolution et efficacité:** l’évolution a été marquée par une très bonne amélioration chez 90% des malades, alors qu'on avait recours au changement du palier chez 10% des malades dont l’évolution a été jugée favorable par la suite. Tous les malades ont été évalués par les mêmes échelles utilisées au départ en plus de l'interrogatoire, l'observation et l'examen clinique.

### Avis des parents concernant la prise en charge de la douleur et le soutien lors des gestes de soins

La prise en charge de la douleur dans le service a été jugée efficace et satisfaisante par 57% des parents. Vingt pourcent (20%) ont souhaité une thérapie meilleure avec des antalgiques plus puissants. Vingt trois pour cent (23%) ont préféré ne pas assister aux actes invasifs.

## Discussion

Tous les patients cancéreux rencontreront, inévitablement, des douleurs au cours de leurs maladies, que celles-ci guérissent ou non. La douleur est un problème courant; une analyse de 32 publications montre que chez 70% des malades ayant un cancer avancé, le symptôme principal est la douleur, et que chez les adultes et les enfants subissant un traitement anticancéreux, jusqu’à 50% d'entre eux ressentent une douleur. Ces douleurs doivent être traitées en priorité comme symptôme d'accompagnement, en analysant les composantes, la physiopathologie, la ou les causes et le mode de survenue. Le traitement de la douleur cancéreuse est un problème de santé publique important, mais négligé; aussi bien dans les pays développé que dans les pays en voie de développement. Il faut insister sur le fait que plusieurs millions de cancéreux qui souffrent chaque jour d'une douleur persistante peuvent être soulagés, si le traitement prescrit est adapté et surveillé selon des règles rigoureuses, afin d'atteindre l'efficacité optimale, grevée des moindres effets secondaires. La prévalence de la douleur chez l'enfant cancéreux en général était l'objet d'une étude italienne de Cornaglia portant sur 814 dossiers d'enfants traités pour cancer entre 1973 et 1982. Elle a retrouvé dans l'histoire du malade des douleurs modérées ou sévères dans 57% des cas [4]. Dans l’étude de MISER [5], la prévalence de la douleur à l'hôpital était de 54%. Dans l'enquête de LJUNGMAN [6] évaluant la prise en charge de la douleur chez l'enfant cancéreux, 60% des enfants se plaignaient de douleurs. Pour GAUVAIN-PIQUARD [7], dans la pratique quotidienne, tous les enfants atteints de cancer feront une expérience douloureuse.

Dans notre étude, la prévalence de la douleur des enfants hospitalisés était de 71,4%, résultat similaire à celui trouvé dans les différentes études. L’évolution de la maladie cancéreuse chez l'enfant fait subir celui ci plusieurs douleurs d’étiologies différentes. Les douleurs liées à l’évolution tumorale: augmentation de la masse tumorale, compression des structures nerveuses, infiltration, inflammation ou surinfection [8–11]. Les douleurs secondaires aux traitements du cancer (la chimiothérapie, la radiothérapie ou la chirurgie): à savoir toxicité locale, digestive, neurologique ou fibrose liée à la radiothérapie: l'ostéoradionécrose, les myélites post-radiques, etc. La répétition des gestes invasifs douloureux génère chez l'enfant une réaction d'anxiété qui, ajouter à la douleur, retentit négativement sur le vécu de la maladie et constitue à distance la principale source de souvenir douloureux. En 1991, Elliot S et Col ont noté, dans une autre étude issue de la Mayo Clinique concernant 160 enfants atteints de cancers surveillés pendant une semaine, que seuls 28 enfants étaient douloureux dont 58% avaient des douleurs liées aux traitements et seulement 21% liées directement au cancer [4]. Dans l’étude de MISER [5], la douleur était surtout induite par les effets secondaires des traitements anti-cancéreux alors que la tumeur n’était à l'origine des douleurs que chez 34.5% des enfants hospitalisés. Dans l'enquête de LJUNGMAN, 49% des douleurs étaient secondaires aux traitements anti-cancéreux, 38% étaient dues aux actes invasifs et 13% uniquement étaient en lien direct avec la maladie cancéreuse. Dans notre étude, la tumeur était à l'origine des douleurs dans 30% des cas, les traitements anti-cancéreux étaient responsables des douleurs dans 30% des cas et 40% étaient secondaires aux actes aigus invasifs à visée diagnostique ou thérapeutique. On constate la prédominance des douleurs dues aux actes invasifs. La localisation des douleurs chez l'enfant cancéreux est variable selon les études, mais parmi les syndromes douloureux les plus fréquemment rapportés figurent les douleurs abdominales, les douleurs liées à une neuropathie périphérique, les céphalées et les douleurs de la cavité buccale [6, 12].

Dans notre étude, les douleurs les plus fréquentes étaient les douleurs abdominales (34%), les douleurs des membres (26%), puis viennent les douleurs de la cavité buccale (représentées essentiellement par les mucites), et les céphalées (20%). La diversité et la multiplicité des douleurs semblent donc habituelles, ces données rendent la prise en charge plus difficile. Dans notre étude, sur les 100 enfants douloureux, la douleur était invalidante (intensité modérée à sévère) dans 87% des cas. Dans l'enquête de LJUNGMAN [6], plus que la moitié des enfants interrogés avaient des douleurs invalidantes. Dans l’étude de COLLINS [13], la douleur était estimée modérée à sévère chez 86,8% des enfants. Ce qui montre la réalité de la souffrance et l'obligation d'un soulagement pour une qualité de vie meilleure. La douleur peut avoir des conséquences variables qu'il faut prendre en considération: des conséquences organiques pouvant être l'origine de véritables cercles vicieux. Des conséquences fonctionnelles à types de troubles neurovégétatifs ou des désordres psychologiques à savoir l'anxiété, la dépression, des troubles de sommeil ou des troubles de l'appétit peuvent également être notées. Dans notre étude, la douleur avait des répercussions négatives sur le sommeil chez la moitié des malades (50%), des troubles de l'humeur chez les deux tiers des malades (66%) et la capacité à jouer avec des enfants ou avec ses propres jouets (74%). LJUNGMAN [6] a objectivé que plus de 50% des enfants avaient des troubles du sommeil. D'où l'importance de chercher et évaluer ces différentes dimensions exprimées par ces enfants pour adapter la prise en charge. Dans notre étude les trente malades dont la douleur était liée à la maladie elle-même, 90% des malades (27 enfants) ont reçu un traitement antalgique, et uniquement 13% (4 malades) ont bénéficié de dérivés morphiniques, et 10% n'ont eu aucun traitement (seuil de la douleur inférieur à 3). Il a été constaté que le traitement instauré initialement chez 16,7% des malades (5 malades) n'a apporté aucune amélioration, ce qui a motivé le changement du palier, les trente malades dont la douleur étaient lié au traitement, 86,6% des malades (26 enfants) ont reçus un traitement antalgique, 23% (7 malades) ont bénéficié de dérivés morphiniques, et 13,4% (4 malades) n'ont eu aucun traitement. Il a été constaté aussi que le traitement instauré initialement n'a apporté aucune amélioration chez 10% des malades, ce qui a motivé le changement du palier. Ce qui montre que ces malades ont reçu un traitement antalgique inadapté à l'intensité de leur douleur.

Des prescriptions inadéquates se retrouvent également dans l’étude de MISER, où un tiers des enfants qui souffraient de la douleur n'ont reçu aucun traitement antalgique, alors que presque la moitié des enfants a bénéficié d'un dérivé morphinique. Dans notre étude, les actes iatrogènes douloureux les plus fréquents étaient les myélogrammes (80%), les ponctions veineuses (60%) et les ponctions lombaires (50%). Dans l'enquête de LJUNGMAN, les injections intramusculaires, sous cutanées et les ponctions veineuses sont les actes les plus douloureux chez respectivement 53%, 30% et 37% des enfants. Par ailleurs, les biopsies ostéo- médullaires étaient très douloureuses pour 86% des enfants qui n'ont pas eu d'anesthésie générale pour la réalisation du geste. On se pose souvent la question sur la présence des parents lorsque L'on réalise un soin à un enfant, et bien souvent on demandera aux parents de sortir pour le bien de l′enfant et le nôtre. On a l′impression que cette présence génère une angoisse chez l′enfant et chez le personnel soignant, et que celle-ci peut donc nuire à la performance du geste. Carbajal a réalisé dans ce sens une étude prospective randomisée auprès de 91 enfants devant subir un geste agressif aux urgences. Il montre que la présence des parents, ne majore l′angoisse ni des enfants, ni des soignants et ne nuit pas à la réussite du geste. Au contraire la présence des parents est un atout majeur dans toutes les situations douloureuses [14–16]. Dans notre étude, la majorité des enfants, ont été accompagnés de leurs parents lors du geste douloureux (64%), représenté dans 95% des cas par la mere, cette presence des parents a été rapportée comme le facteur qui aidait plus les enfants à supporter la douleur. Quarante pourcent (40%) des enfants ont été informés sur la potentialité de la douleur du geste, dans 30% des cas l'information a été communiquée directement aux parents, alors que chez 30% des malades aucune information concernant l'acte n'a été communiquée. Dans une étude sur le tempérament sensible à la douleur chez des enfants cancéreux, il a été observé que les plus hauts niveaux de sensibilité à la douleur était associés a une anxiété et une douleur plus grande, à la fois pendant et après une ponction lombaire [17]. L'information de l'enfant sur la nature et l'intensité de la douleur de l'acte, permettent une préparation psychologique (interventions cognitivo- comportementales) et d’établir un climat de confiance pour une prise en charge bien codifiée [18–20].

## Conclusion

La douleur chez l'enfant cancéreux est un phénomène complexe, auquel nous sommes confrontés dans notre pratique quotidienne, sa prise en charge nécessite un respect des recommandations internationales, mais surtout des efforts consentis de la part de tous les intervenants à différentes étapes, allant du diagnostic et l'annonce de la maladie à la prise en charge thérapeutique. Les résultats de notre étude montrent une amélioration de la prise en charge de la douleur chez l'enfant atteint du cancer au service de pédiatrie, cette amélioration ne peut être continuée que par la recherche systématique du syndrome douloureux, son évaluation, et l'intégration de tout le personnel soignant en collaboration avec les parents, pour aboutir à une standardisation des attitudes thérapeutiques, dont le but essentiel est de préserver la qualité de vie des enfants cancéreux.
